# Aberrant expression of AKR1B1 indicates poor prognosis and promotes gastric cancer progression by regulating the AKT-mTOR pathway

**DOI:** 10.18632/aging.205041

**Published:** 2023-09-24

**Authors:** Luojie Liu, Lihua Zhu, Zhengwu Cheng, Yibin Sun, Yuqing Zhou, Jiwei Cao

**Affiliations:** 1 Department of Gastroenterology, Changshu Hospital Affiliated to Soochow University, Suzhou 215501, China; 2 Department of Gastrointestinal Surgery, The First Affiliated Hospital of Wannan Medical College, Wuhu 241000, China; 3 Department of Gastroenterology, The Affiliated Suzhou Hospital of Nanjing Medical University, Suzhou Municipal Hospital, Gusu School, Nanjing Medical University, Suzhou, Jiangsu 215005, China; 4 Department of General Surgery, The Affiliated Suzhou Hospital of Nanjing Medical University, Suzhou Municipal Hospital, Gusu School, Nanjing Medical University, Suzhou, Jiangsu 215005, China

**Keywords:** AKR1B1, gastric cancer, prognosis, AKT-mTOR pathway, proliferation and migration

## Abstract

Gastric cancer (GC) is a common malignant tumor in the digestive tract and a major cause of global cancer death. Due to the limited access to early screening, many patients are diagnosed with advanced GC. Therefore, postoperative radiotherapy and chemotherapy possess limited efficacy in treating GC. AKR1B1 has been associated with tumorigenesis and metastasis across various tumors, becoming a potential therapeutic target for GC. However, its role and mechanism in GC remain unclear. In this study, AKR1B1 was elevated in GC tissue, depicting a poor prognosis. AKR1B1 is closely related to age, vascular and neural invasion, lymph node metastasis, and the TNM stage of GC. The developed survival prediction model suggested that AKR1B1 expression level is crucial in the prognosis of GC patients. Moreover, the expression level of AKR1B1 in GC tissues is closely associated with the AKT-mTOR pathway. *In vitro* and *in vivo* assays functional assays helped determine the oncogenic role of AKR1B1. Additionally, the knockdown of AKR1B1 expression level in GC cell lines could effectively suppress the AKT-mTOR pathway and inhibit the proliferation and migration of tumor cells. In conclusion, this study provides a theoretical basis to establish the potential association and regulatory mechanism of AKR1B1 while offering a new strategy for GC-targeted therapy.

## INTRODUCTION

Gastric cancer (GC) is a common malignant tumor in the digestive tract and one of the major causes of global cancer death. [1, 2]. The GC incidence is higher in Asia due to geographic differences, Helicobacter pylori infection, and lifestyle factors, including smoking and alcohol consumption. In China, many GC patients have not been actively screened at an early stage, leading to advanced tumor stages when confirmed [3, 4]. In recent years, continuous progress in surgical techniques and treatment method diversification have effectively elevated survival rate of GC sufferers. Nevertheless, there is a bottleneck in improving the overall treatment effect. Therefore, the key steps to treat GC involve identifying effective diagnostic and therapeutic targets, developing prognostic prediction models, and enhancing therapeutic effects.

Aldo-keto reductases (AKRs) are oxidoreductases which rely on nicotinamide adenine dinucleotide phosphate (NADPH) and are the most glucose reductases. [5, 6]. Aldo-keto reductase 1 member B1 (AKR1B1) is an AKR family member. The space structure of AKR1B1 protein is a single-chain polypeptide, generally existing as a monomer among living biosome. AKR1B1 is able to reversibly bind to NADPH, and the AKR1B1 protein is widely distributed across the human body and is expressed in various tissues and organs [7–9].

The aberrant AKR1B1 protein expression in human tissues has been related to the initiation and progression of various chronic diseases and cancers [7–9]. Studies have demonstrated a causal association between AKR1B1 activity and diabetic complications, such as cataracts, retinopathy, neuropathy, and nephropathy [10, 11]. These chronic diabetes complications are the principal reason for disability or death in such sufferers. In addition, the expression quantity and activity of AKR1B1 protein in human lung cancer, alcoholic liver disease, liver cancer, and breast cancer were significantly elevated and were closely related to tumor progression [6, 9, 12]. Therefore, we focused on the expression changes of AKR1B1 during the occurrence and progression of GC, its relevance with clinical survival and prognosis, and the impact of its aberrant expression on the hyperplasia and metastasis of tumor cells. Thus, our study could provide a new GC-targeted therapeutic strategy with a strong theoretical basis to target AKR1B1 in GC.

## MATERIALS AND METHODS

### Specimen collection

Our study recruited 115 patients with primary GC and paired contiguous normal stomach tissues after undergoing radical gastrectomy from the First Affiliated Hospital of Wannan Medical College during 2015–2016. Each subject participating in our study signed written informed consent forms. The agreement followed the ethical guidelines set out in the Declaration of Helsinki. The Affiliated Suzhou Hospital of Nanjing Medical University (ID: 2021240) and the institutional review board of the First Affiliated Hospital of Wannan Medical College (ID: 202248) approved this study.

### Immunohistochemistry

We fixed the GC specimens and paired contiguous normal stomach tissues with formalin. Then, they were buried in paraffin and cut into 5-μm slices for immunohistochemical staining as referred in a previous study [13]. Sections were cultured at indoor temperature for 2 hours using 1:100 diluted anti-AKR1B1 antibody (1:100, Bioss, China). Two blinded researchers evaluated the staining scores to analyze immunohistochemistry (IHC) results. IHC score was decided by the multiple of the intensity (0 as negative; 1 as weak; 2 as moderate; 3 as strong) and extent (0 as 0–5%; 1 as 6–25%; 2 as 26–50%; 3 as 51–75%; 4 as >75%) score. The final point of 0 was considered −; 1–4 considered +; 5–8 considered ++; 9–12 considered +++. In our research, ++ or +++ was deemed to be a positive protein expression, and – or + was considered to be negative.

### Cell cultures

The included human GC cell strains were acquired from the Nanjing Medical University (Nanjing, China). They were sprouted in RPMI medium 1640 (Hyclone, USA) with 10% FBS (Gibco, USA), 100 units/ml penicillin G sodium, and 100 μg/mL streptomycin sulfate (Gibco, USA). The clones were cultivated in 5% CO_2_ at 37°C.

### Transfection with shRNA

These cells were cultured until 80% fusion and transfected through a lentivirus AKR1B1-shRNA technology (Human AKR1B1-targeting shRNA were procured from GenePharma, China) according to the company’s manuals [14]. The cells after transfection were screened using G418 (Roche, Switzerland). Additionally, cell lines with a stable AKR1B1 knockdown were picked for subsequent research.

### Protein preparation and western-blot test

The whole proteins were extracted with RIPA lysis buffer comprising proteinase and phosphatase inhibitors (Sigma, USA). The whole protein was separated with SDS-PAGE and shifted to a PVDF membrane which was sealed using 5% skimmed milk and probed using antibodies at 4°C all night long. We visualized the protein bands using chemiluminescence analysis and quantified them with ImageJ (NIH, USA). Antibodies comprised in western-blot were anti-AKR1B1 (1:1000, Bioss, China), anti-p-AKT (1:1000, Cell Signaling Technology, USA), anti-p-S6K1 (1:1000, Cell Signaling Technology, USA), and anti-β-actin (1:1000, Bioss, China) antibodies.

### Colony-forming test

About a thousand cells were cultivated in every 6 pore plates for 10 days. The poietic cell lines were fixed by methanol and dyed with 0.1% crystal violet solution. Finally, they were calculated using an optical microscope (Nikon, Japan) equipped with a digicam (Nikon, Japan).

### Transwell test

Cells were inoculated into the upper cavities at a density of 10,000 cells/200 μl in a serum free medium. Each well in the lower cavities was full of complete medium of 800 μl. After incubation for twelve hours, the filters were fixed using 4% paraformaldehyde and stained by 0.1% crystal violet, and the mean value of 5 random fields in each sample was determined.

### Cell viability assay

The GC cell viability was measured with the Cell Counting Kit-8 (CCK-8) test (Abmole Bioscience, USA) in 96-well plates with 2000 cells/well based on the manufacturer’s protocols.

### Subcutaneous xenograft

SPF male BALB/c naked mice (four weeks, approximately 19 g) were stochastically split into groups, each group of 5. After one week of adaptation, 10^7^ AKR1B1-KD or NC-shRNA AGS cells were injected to the right dorsal subcutaneous area of the mice on day 0. Then we measured the weight of mice fortnightly, and the dimensions of their subcutaneous neoplasms was determined. Four weeks later, the subcutaneous neoplasms were peeled and weighed. Ethical clearance by the Animal Ethics Committee of the Affiliated Suzhou Hospital of Nanjing Medical University (Suzhou, China) has been obtained for all the experiments in this study.

### Statistical analysis

The data were shown as mean ± S.D. of a minimum of three stand-alone experiments. Data were analyzed using SPSS 22.0, GraphPad Prism 8, and R software (version 3.6.1). Two groups were compared using the unpaired and two-tailed *t*-test or Mann–Whitney *U* test. The IHC results were analysed using Chi-squared or Fisher's exact tests. *P* < 0.05 was determined statistically significant.

### Data availability

Data will be made available on request.

## RESULTS

### Increased AKR1B1 expression in GC tissues and their association with clinicopathological indicators

**Figure 1:**
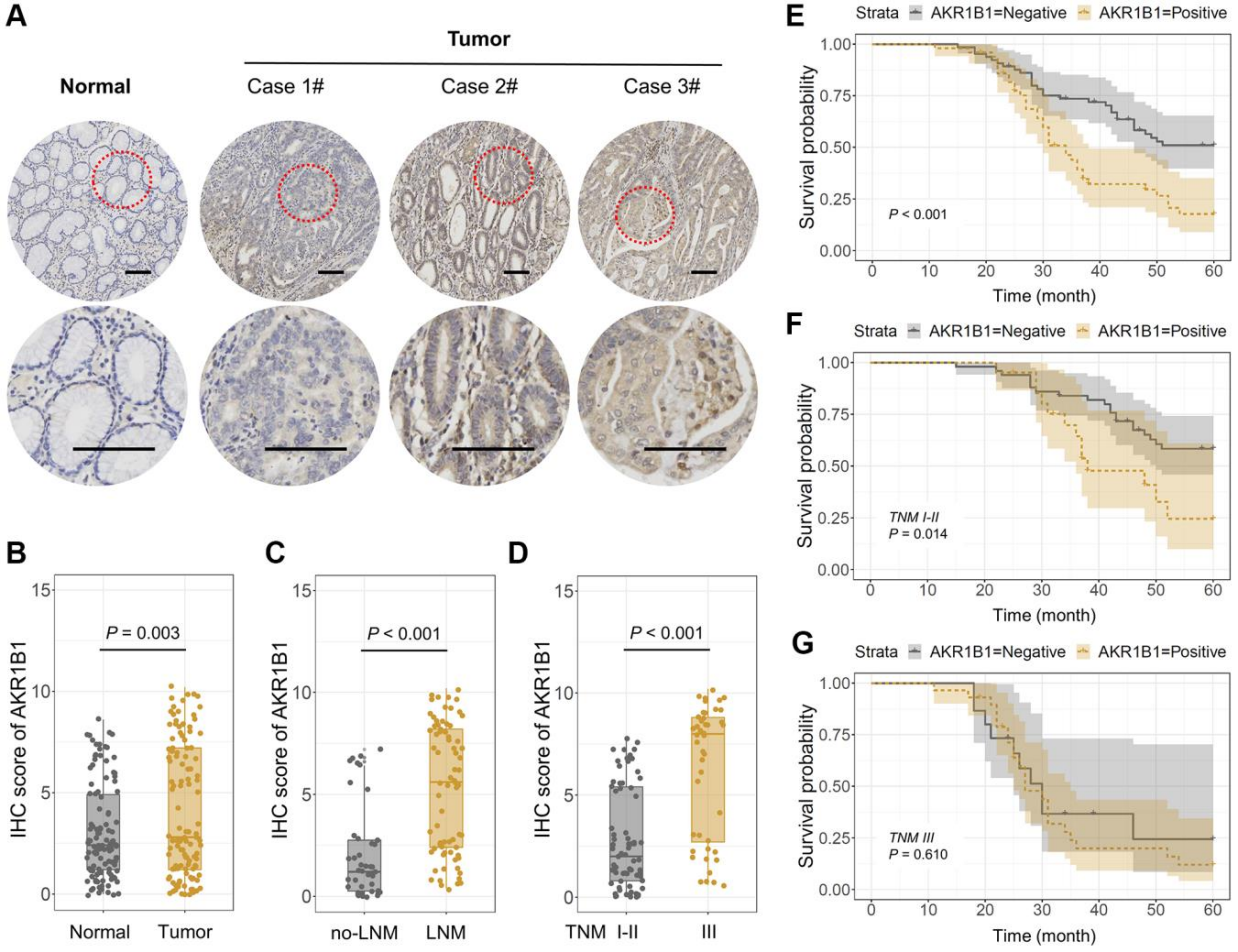
**AKR1B1 expression in GC and adjacent normal tissues.** (**A**) The representative IHC images depicting *in situ* AKR1B1 expression in Case 1#, 2#, 3# GC and adjacent normal tissues (scale bar = 100 μm). (**B**–**E**) The IHC scores of AKR1B1 in (**B**) GC vs. normal tissues, (**C**) Tumors with or without lymph node metastasis, and (**D**) TNM stage I–II vs. stage III. (**E**) The overall survival analysis of AKR1B1^pos^ vs. AKR1B1^neg^ GC patients. (**F**) In the subgroup of TNM staging I–II, the overall survival analysis of AKR1B1^pos^ vs. AKR1B1^neg^ GC patients. (**G**) The overall survival analysis of AKR1B1^pos^ vs. AKR1B1^neg^ GC patients in the subgroup of TNM staging III.

Firstly, the AKR1B1 expression was detected in 115 GC tissues and contiguous normal stomach tissues using IHC. The IHC points were decided based on the intensity and extent of the staining (Figure 1A). The positive AKR1B1 expression levels among normal and cancer gastric cancer tissues were 43.5% and 26.1%, respectively. Based on the IHC scores, there was a high expression of AKR1B1 in GC tissues in contrast to contiguous normal stomach tissues (*P* = 0.003, Figure 1B). What’s more, AKR1B1 expression was observably elevated in patients with lymphaden infiltration (*P* < 0.001, Figure 1C) and TNM classification III (*P* < 0.001, Figure 1D).

The staining results were divided into negative and positive based on the IHC score to determine the AKR1B1 expression and its prospective influence in GC tissues. The pertinence between AKR1B1 expression within GC tissues and the clinical pathological indexes of GC patients was assessed. The results suggested AKR1B1 expression was observably correlated with age (*P* = 0.044), vascular invasion (*P* = 0.030), neural infiltration (*P* = 0.017), lymphaden metastasis (*P* < 0.001), and the TNM classification (*P* < 0.001, Table 1). In contrast, no pertinence could be detected between AKR1B1 expression and different clinical pathological indexes (*P* > 0.05, Table 1).

**Table 1:** Association between AKR1B1 and clinic-pathological factors in GC patients.

**Variables**	**AKR1B1**		
	**Negative**	**Positive**	***P* value**
Age (years)			
<65	28	31	0.044^a^
≥65	37	19	
Gender			
Male	43	37	0.365
Female	22	13	
Tumor size (cm)			
<5	48	36	0.825
≥5	17	14	
Degree of differentiation			
Well	23	11	0.119
Poor	42	39	
Vascular invasion			
Negative	43	23	0.030^a^
Positive	22	27	
Neural invasion			
Negative	38	18	0.017^a^
Positive	27	32	
Depth of tumor invasion			
T1-2	27	13	0.083
T3-4	38	37	
Lymph node metastasis			
No	34	8	<0.001^b^
Yes	31	42	
TNM staging			
I–II	50	21	<0.001^b^
III	15	29	

^a^*P* < 0.05, ^b^*P* < 0.001.

### Aberrant expression of AKR1B1 in GC tissues indicates low survival rate

Depending on the close connections between AKR1B1 and clinicopathological indicators, the influence of AKR1B1 expression level was further assessed in GC tissues, affecting the overall survival rate of sufferers. The patients were classified based on AKR1B1^pos^ (AKR1B1 positive) and AKR1B1^neg^ (AKR1B1 negative) expressions. The survival rate of AKR1B1^pos^ sufferers was obviously lower than AKR1B1^neg^ sufferers (*P* < 0.001, Figure 1E). Further subgroup analysis in the light of TNM classification indicated that high AKR1B1 expression determined the low survival rate of sufferers in the stage I-II subgroup (*P* = 0.014, Figure 1F). Nevertheless, AKR1B1 expression was found to be statistically unrelated to overall survival rate in the stage III subgroup (*P* = 0.610, Figure 1G). Thus, AKR1B1 expression could only predict the prognosis of early-stage GC patients instead of an advanced stage.

**Figure 2:**
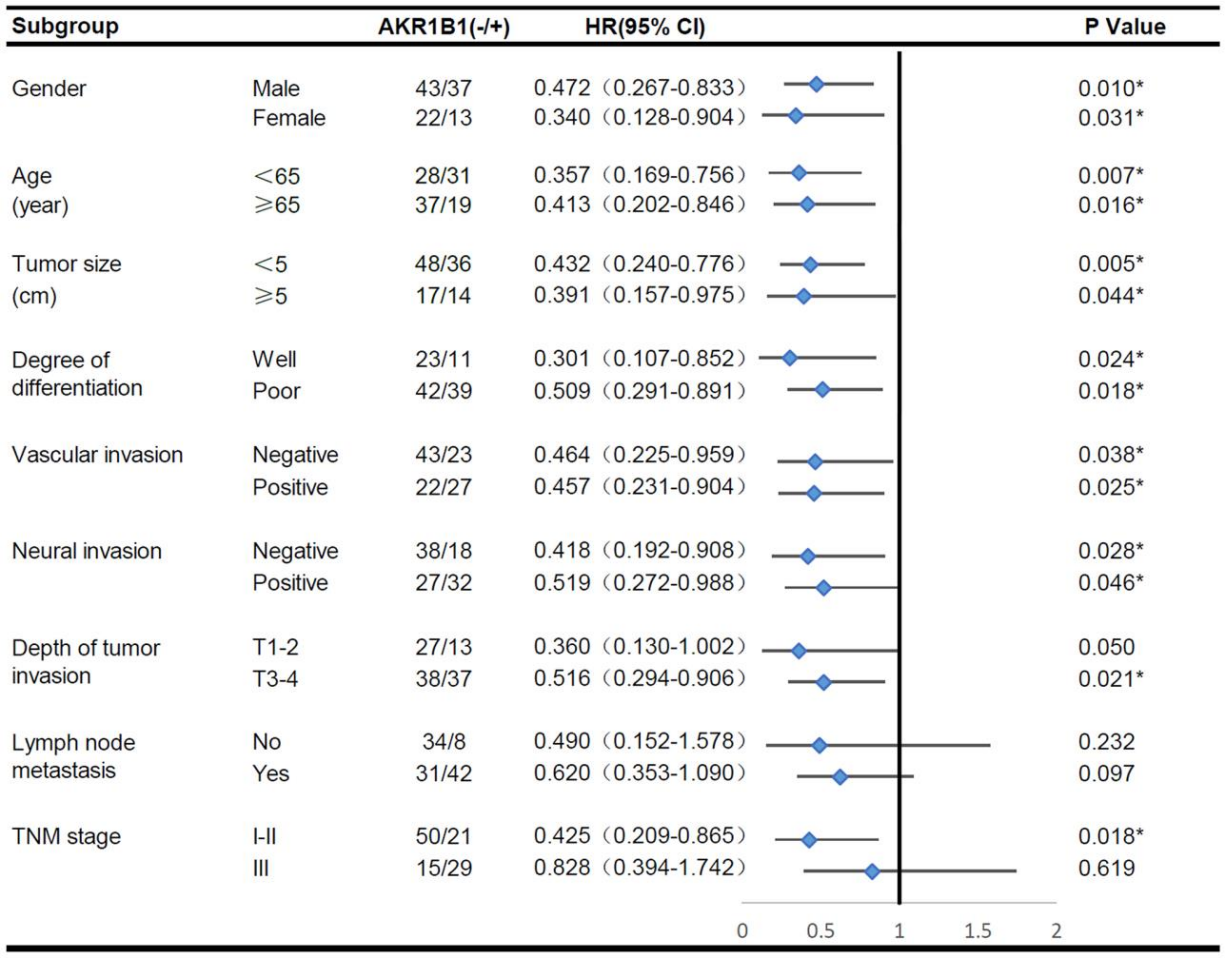
The overall survival analysis of AKR1B1^pos^ vs. AKR1B1^neg^ GC patients in subgroups has been demarcated based on individual clinicopathological indicators.

A subgroup survival analysis was performed according to additional clinical pathological indexes to clarify the predictive effect of AKR1B1 expression on survival across different clinicopathological subgroups. The survival rate of AKR1B1^pos^ patients was markedly lower than AKR1B1^neg^ patients (*P* < 0.05, Figure 2), irrespective of gender, age, differentiated degree, the size of tumor, and vascular and perineural infiltration. Intriguingly, the AKR1B1 expression level did not significantly affect the tumor invasion depth T1-2 (*P* = 0.050) or patients with TNM stage III (*P* = 0.618). Conversely, increased AKR1B1 expression was related to worse prognosis in GC patients with the tumor invasion depth T3-4 (*P* = 0.021) or TNM stage I-II (*P* = 0.018). Meanwhile, high AKR1B1 expression could not predict a significant effect on survival, irrespective of whether they belonged to lymph node (*P* = 0.097) or no lymphaden (*P* = 0.232) metastases. Consequently, the AKR1B1 expression level can be detected in GC tissues to evaluate patient survival depending on the clinicopathological indicators.

**Figure 3:**
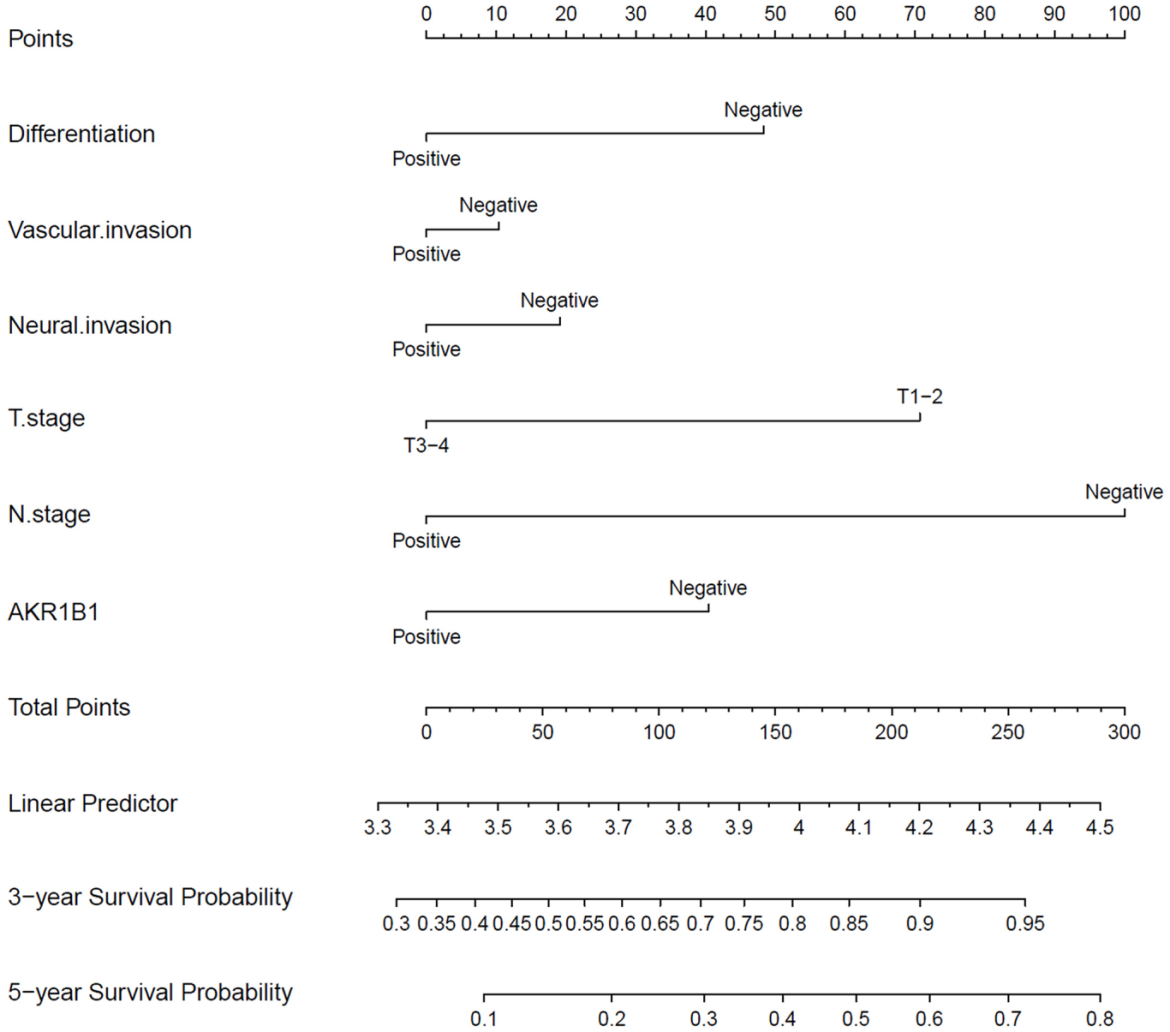
**The nomograms for predicting 3- and 5-year overall survival of GC patients.** The points of each variable were obtained using a vertical line between each variable and the point scale. The predicted survival rate was connected with the total points through a vertical line drawn from the total points scale to the overall survival.

The single factor analysis of Cox’s proportional risk model proved that differentiation degree, vascular invasion, neural invasion, tumor invasion depth, lymphaden metastasis, and AKR1B1 expression (*P* < 0.05, Table 2) are prognosis factors affecting GC patient survival. Lymph node metastasis acted as the independent factor in the multivariate analysis. Moreover, other indicators, such as AKR1B1 expression, were important in prognosis. By nomograms, we proved the pivotal part of the AKR1B1 expression level in GC to predict post-operative patient survival (Figure 3). The nomograms could forecast the overall survival rate of GC sufferers at 3 and 5 years. The negative or positive AKR1B1 expression could significantly affect the total points, demonstrating the pivotal part of AKR1B1 in forecasting GC patient survival (Figure 3).

**Table 2:** Results of univariate and multivariate analyses of postoperative patients’ survival by Cox’s proportional hazard model.

**Varieties**	**Univariate analysis**			**Multivariate analysis**		
	**HR**	**95% CI**	***P***	**HR**	**95% CI**	***P***
Age (≤60 or >60 years)	0.913	0.563–1.479	0.711			
Gender (male/female)	1.349	0.777–2.345	0.288			
Size of tumor (≤5 or >5 cm)	0.718	0.424–1.214	0.216			
Degree of differentiation (moderate-well/poor)	0.521	0.292–0.929	0.027^a^	0.650	0.361–1.170	0.151
Vascular invasion (negative/positive)	0.424	0.260–0.691	0.001^b^	0.746	0.432–1.287	0.293
Neural invasion (negative/positive)	0.433	0.262–0.716	0.001^b^	0.716	0.396–1.296	0.269
Depth of tumor invasion (T1-2/T3-4)	0.360	0.201–0.645	0.001^b^	0.631	0.314–1.269	0.196
Lymph node metastasis (negative/positive)	0.265	0.146–0.482	<0.001^c^	0.437	0.219–0.873	0.019^a^
AKR1B1 expression (negative/positive)	0.420	0.257–0.686	0.001^b^	0.651	0.388–1.093	0.104

^a^*P* < 0.05, ^b^*P* < 0.01, ^c^*P* < 0.001.

### Ectopic AKR1B1 regulates the AKT-mTOR pathway to facilitate GC cell migration and proliferation

**Figure 4:**
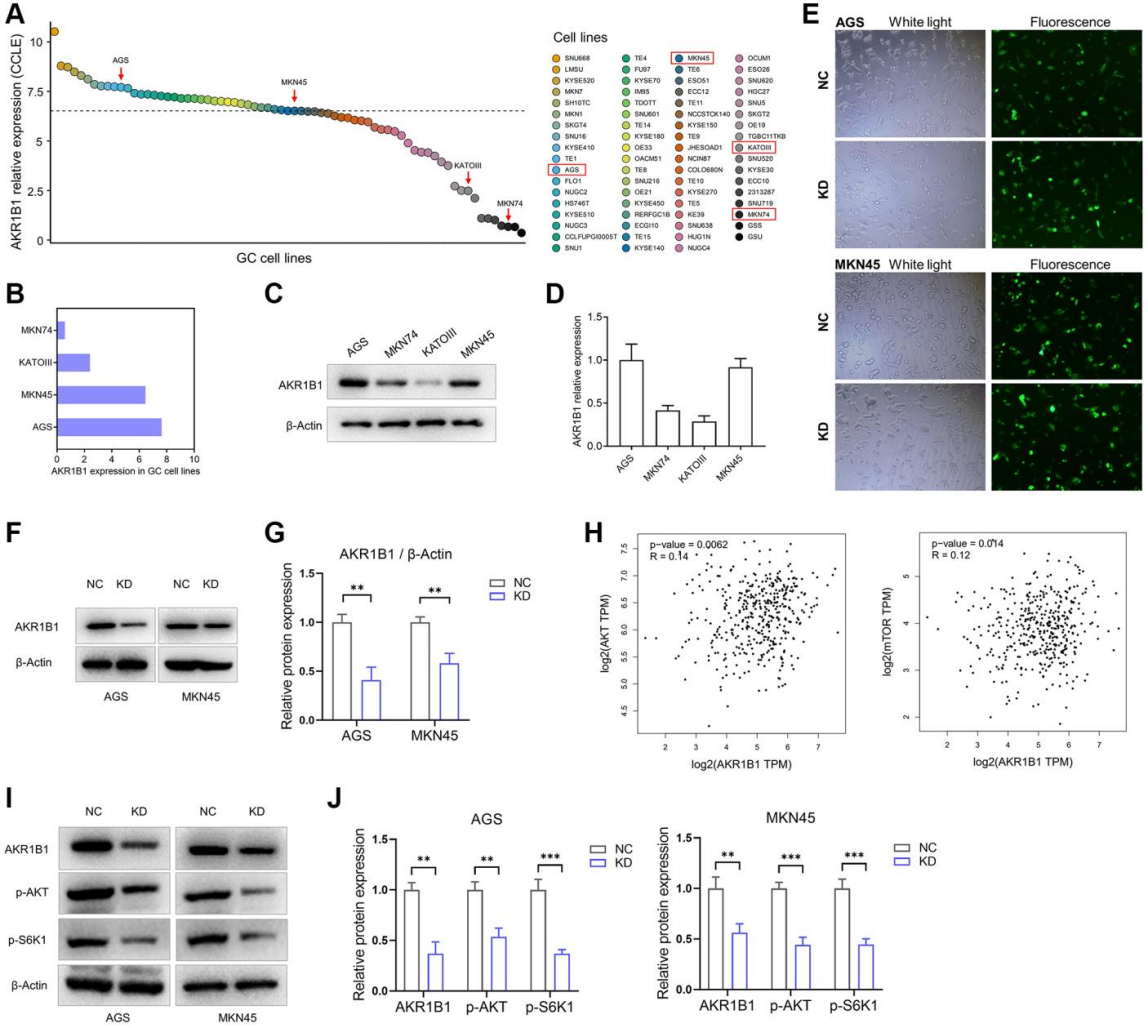
**AKR1B1-KD cells were constructed, and the regulatory relationship between AKR1B1 and AKT-mTOR pathway was evaluated.** (**A**) The AKR1B1 expression in GC cell lines was searched from the CCLE platform. (**B**) The CCLE platform helped select AKR1B1 expression in four GC cell lines. (**C**) AKR1B1 expression in four GC cell lines was detected using western blotting. (**D**) The immunoblot result of AKR1B1 expression was semi-quantified in four GC cell lines with ImageJ. (**E**) The white and fluorescence microscope images of AGS and MKN45 cells were transfected with control-shRNA (NC) and AKR1B1-shRNA (KD). (**F**) Western blot indicates the AKR1B1 protein levels in NC and AKR1B1-KD AGS and MKN45. (**G**) The immunoblot results were semi-quantified using ImageJ. (**H**) The GEPIA platform was used to determine the correlation analysis of *AKR1B1* and *AKT* or *mTOR* gene expression levels in GC patients from TCGA datasets. (**I**) Western blot reveals the AKR1B1, p-AKT, p-S6K1, and β-actin protein levels in AKR1B1-KD GC cells. (**J**) The immunoblot results of AGS were semi-quantified using ImageJ. Abbreviations: CCLE: Cancer Cell Line Encyclopedia; NC: negative control; KD: AKR1B1-shRNA. ^**^P < 0.01, ^***^P < 0.001.

To gain mechanistic insights into the AKR1B1 effects on GC cell migration and proliferation and the prospective regulatory mechanism of the AKT-mTOR pathway, we firstly retrieved AKR1B1 expression in GC clones through the Cancer Cell Line Encyclopedia (CCLE) (Figure 4A, 4B). Whereafter, AKR1B1 expression was detected in GC clones using western blotting, consistent with CCLE platform data (Figure 4C, 4D). Therefore, AGS and MKN45 were selected with high expression of AKR1B1 for transfection with AKR1B1-shRNA and establishment of AKR1B1 knockdown cell lines (AKR1B1-KD). The fluorescence microscope images of AGS and MKN45 cells with control-shRNA and AKR1B1-shRNA transfection were detected to evaluate the knockdown effect (Figure 4E). We also investigated the effect of AKR1B1-KD GC cells with immunoblot, and AKR1B1 knockdown was evident in GC clones (Figure 4F, 4G).

The prospective relevance between AKR1B1 and the AKT-mTOR pathway was analyzed in TCGA datasets using the GEPIA database. The expression degree of *AKR1B1* in GC tissues had a tight connection with the expression levels of *AKT* (*P* = 0.006) and *mTOR* (*P* = 0.014, Figure 4H). The expression of phosphorylated S6K1 in cancers depicted the AKT-mTOR pathway activation status as a key downstream target. This pathway is crucial in the migration, proliferation, apoptosis, drug resistance, and various abilities of cancer cells [15, 16]. Consequently, we explored the relationship between AKR1B1 expression and phosphorylated AKT and S6K1 using AKR1B1-KD GC cell lines. The outcome was inhibited in AKR1B1-KD GC clones, p-AKT, and p-S6K1 levels (Figure 4I, 4J).

**Figure 5:**
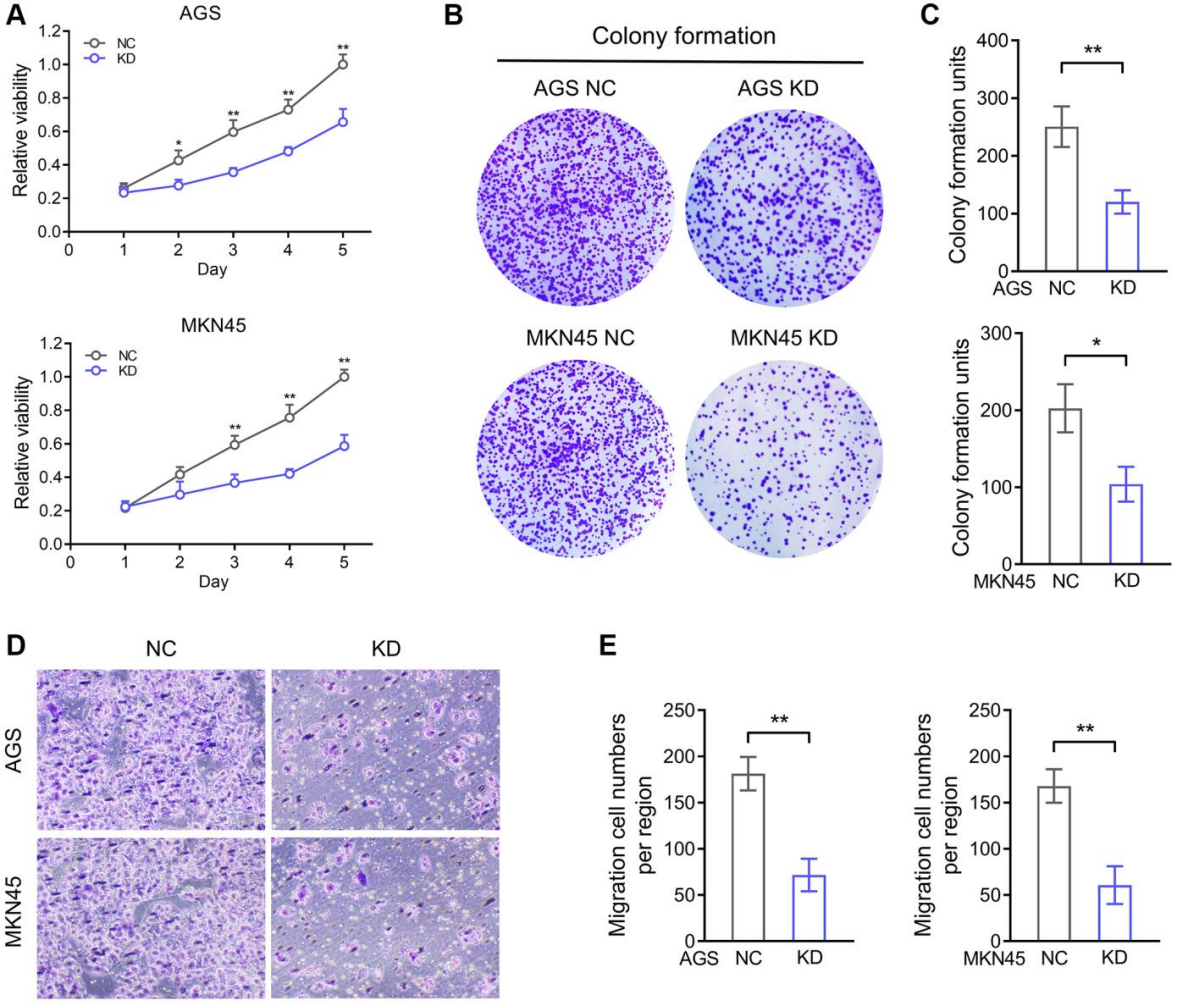
**AKR1B1 enhances the proliferation and migration ability of GC cells.** (**A**) The CCK-8 assay determined the proliferation ability of AGS and MKN45 transfecting with AKR1B1-shRNA. (**B**) The colony formation capacity of AGS and MKN45 transfected with AKR1B1-shRNA was assessed. (**C**) The colony-forming units were counted. (**D**) The migration capacity of AGS and MKN45 transfected with AKR1B1-shRNA was assessed. (**E**) The number of migrating cells was counted. Abbreviations: NC: negative control; KD: AKR1B1-shRNA. ^*^P < 0.05, ^**^P < 0.01.

The biology function of AKR1B1 was analysed by AGS and MKN45 knockdown structures. We investigated the multiplication capacity of AKR1B1-KD cells through the CCK-8 and colony-forming assays. Compared with negative controls, the hyperplasia of AKR1B1-KD cells was more markedly repressed (Figure 5A). Agree with these results, the AKR1B1-KD cells similarly exhibited a poor colony-forming capacity (Figure 5B, 5C). Then, the migration capacity alteration of the AKR1B1-KD cells was evaluated using a transwell assay, which was markedly suppressed (Figure 5D, 5E).

### AKR1B1 promoting tumorigenesis and deteriorating nutrition condition *in vivo*

**Figure 6:**
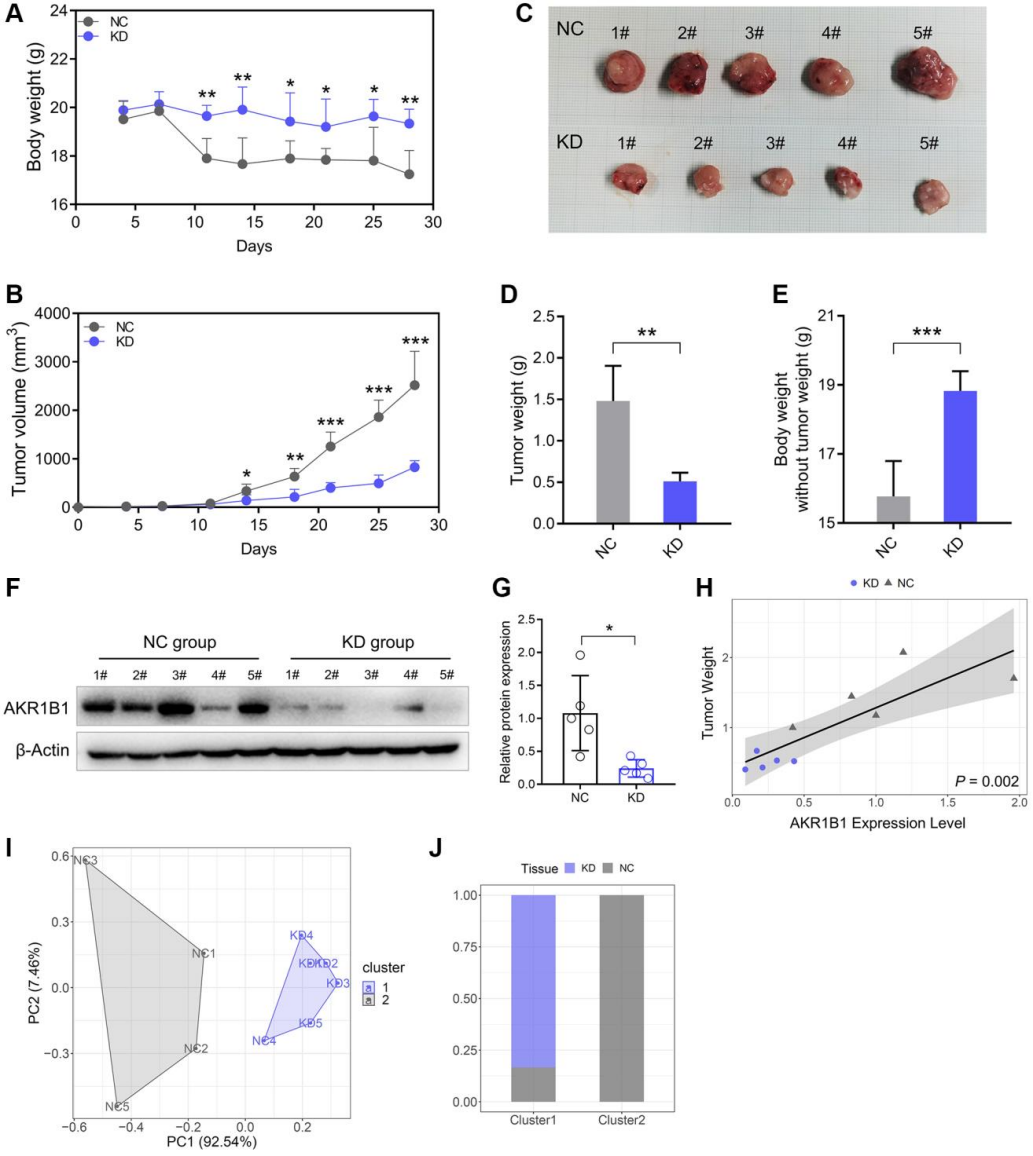
**Reduced AKR1B1 inhibits GC tumor growth *in vivo*.** (**A**) During the experiment, the body weight of mice was recorded twice a week. (**B**) The tumor volume of mice was recorded twice a week during the experiment. (**C**) The representative images of subcutaneous tumors harvested from NC and AKR1B1-KD groups. (**D**) The weights of tumor masses. (**E**) Net body weight after subtracting the respective tumor weights. (**F**) AKR1B1 expression was detected in subcutaneous tumors using western blotting. (**G**) The immunoblot result of AKR1B1 expression in subcutaneous tumors was semi-quantified using ImageJ. (**H**) The association analysis between the AKR1B1 expression levels in tumor tissues and weight. (**I**) The stratification of mice in Cluster 1 and Cluster 2 depends on the tumor weight and AKR1B1 expression levels. (**J**) The percentage of NC and AKR1B1-KD mice in each cluster. *n* = 5 for each group. Abbreviations: NC: negative control; KD: AKR1B1-shRNA. ^*^P < 0.05, ^**^P < 0.01, ^***^P < 0.001.

We built a xenograft murine model using AGS cell lines to demonstrate the AKR1B1 expression level influences on neoplasm growth and nutrition in mice. In contrast to the NC group, the nutrition condition of mice was markedly improved in the AKR1B1-KD group (Figure 6A). Simultaneously, the growth of subcutaneous neoplasms was markedly lowered in the AKR1B1-KD group than that in the NC group (Figure 6B, 6C). The mouse weight was observably influenced by the growth of subcutaneous neoplasms. Additionally, AKR1B1 expression had a vital impact on GC cell hyperplasia and the nutrition condition of mice. Therefore, the weights of tumor masses were investigated to calculate the net body weight after deducting the respective tumor weights. Observably inhibiting the proliferative capacity of GC cells in the AKR1B1-KD group appeared as reduction of tumor weight and reverse of mouse weight without tumor weight compared with the control group (Figure 6D, 6E).

Furthermore, the AKR1B1 protein expression was detected in subcutaneous tumors with immunoblot. Thus, AKR1B1 expression was more significantly inhibited in the AKR1B1-KD group than in the control group (Figure 6F, 6G). AKR1B1 protein levels were prominently correlated *in situ* with the subcutaneous neoplasm weight (Figure 6H). Then, the cluster analysis was performed based on the neoplasm weight and AKR1B1 protein levels. The influence of AKR1B1 expression degree was evaluated on the neoplasm growth in mice (Figure 6I). The results indicated that 20% of the NC group mice and 100% of the AKR1B1-KD group mice belonged to Cluster 1. Moreover, 80% of the NC group mice belonged to Cluster 2 (Figure 6J).

## DISCUSSION

GC is a malignant digestive tract tumor possessing high morbidity and mortality [17, 18]. Many patients in China are diagnosed with advanced tumors since active early screening in GC patients is lacking. In recent years, the treatment of GC has considerably improved with the continuous progress of surgical techniques and the diversification of therapeutic means. However, poor surgical effects and serious chemotherapy resistance are still unavoidable problems for many advanced GC patients [19–21]. Therefore, identifying new targets for diagnosis and treatment while constructing new diagnosis and treatment prediction models are effective strategies for treating GC.

Most AKR family members can perform redox reactions using NADPH, a niacinamide adenine dinucleoside phosphate, as the cofactor. Each AKR family member is a soluble protein with a molecular weight of 34–37 kDa. There are 16 families with more than 190 members, widely distributed in the prokaryotes and eukaryotes [6, 22]. Meanwhile, many substrates are also present, including chemical carcinogens, fatty aldehydes, carbohydrates, ketone steroids, ketone prostaglandins, and so on. Each family member is similar in protein folding mode with significantly different function [23].

Studies of AKR1B1, an AKR family member, have focused more on diabetes, primarily its association with disease development and complications. Researchers have obtained many AKR1B1 protein inhibitors using biological metabolite extraction or chemical synthesis to utilize them as new clinical drugs for treating diabetic complications [24–26]. In recent years, AKR1B1 studies have focused on tumorigenesis correlation. Researchers observed that AKR1B1 expression was aberrantly increased in malignant tumors, and its expression level affected the survival prognosis of patients [6, 9, 12]. In colorectal carcinoma, the AKR1B1 and AKR1B10 expression levels in tumor cells, both AKR family members, and their effect on the proliferation ability of tumor cells showed opposite trends. AKR1B10 was significantly reduced in tumor tissues and cells, and its overexpression could effectively inhibit tumor growth. However, AKR1B1 expression promotes tumor cell proliferation with the colorectal cancer progression [27].

Previous studies have demonstrated that AKR1B1 expression is elevated in GC tissues in contrast to contiguous normal stomach tissues [28]. We performed immunohistochemical staining on 115 GC tissues and paired contiguous normal tissues, and the experimental data showed that AKR1B1 expression was anomalously increased in GC tissues in contrast to paired normal stomach tissues. Furthermore, the AKR1B1 expression level was observably higher in GC tissues of patients with lymphaden metastasis positive or TNM III than those with no lymph node spread or TNM I-II. Therefore, the expression of AKR1B1 in cancerous tissues was elevated with GC tumorigenesis. After tumor formation, the AKR1B1 expression level was further increased with disease progression, which was higher than in early-stage GC tissues. Thus, the progression of GC can be assessed based on AKR1B1 expression. The relationship between AKR1B1 expression and GC progression demonstrated that AKR1B1 can potentially promote the invasion and metastasis of cancerous cells in GC patients.

Additionally, by the correlation analysis between the IHC test of AKR1B1 in GC tissues and the clinical pathological data of GC patients, the AKR1B1 expression was tightly related to the age, lymphaden metastasis, vascular invasion, perineural invasion, and TNM classification of patients. The Kaplan-Meier survival analysis depicted that overexpression of AKR1B1 forecasted poor survival outcomes. Interestingly, the results also demonstrated that high expression of AKR1B1 forecasted poor outcomes in TNM I-II patients. In contrast, AKR1B1 expression had nothing to do with prognosis in TNM III patients. This result was further confirmed in the Cox survival subgroup analysis, depicting the accurate application range of AKR1B1 as a prognostic predictor.

The 3-year or 5-year nomogram analysis depicted that AKR1B1 expression markedly influenced the overall survival rate of GC patients, providing a theoretical foundation for targeted AKR1B1 treatment. The aberrant AKR1B1 expression can potentially affect the biological behavior of tumor cells [29–31]. It has been found that the expression level of AKR1B1 was connected with the EMT process of mammary cancer cells. Thus, AKR1B1 methylation detected can become a marker for the early breast cancer diagnosis [32]. DNA chip data helped analyze the gene expression profile of drug-resistant hepatocellular carcinoma from nodules to tumor models, indicating the upregulation of the AKR1B1 expression [33]. While studying colorectal cancer using gene enrichment analysis, tumor genome atlas, and RNAseq data, AKR1B1 expression was associated with cell cycle progression, movement, and inflammation [8, 27]. Studies investigating the correlation between AKR1B1 and lung cancer revealed that the AKR1B1 expression change could be significant in the lung cancer metastasis [9]. In addition, AKR1B1 inhibition may play the role of an auxiliary therapy rendering tumor cells more sensitive to anti-tumor therapy or alleviating the untoward reactions [34, 35].

The expression of AKR1B1 was knocked down, and the changes in the migration and proliferation ability of GC cells were analyzed to explore the function of AKR1B1 in GC cells. The findings indicated that the migration and proliferation ability of GC cells was obviously inhibited. We also conducted relevant experiments *in vivo* by constructing subcutaneous tumor model of mice. The tumor was induced by subcutaneous injection of AKR1B1-KD or NC cells. The subcutaneous neoplasm of the AKR1B1-KD group was markedly smaller than that of the NC group. Moreover, the nutrition condition of the AKR1B1-KD group was significantly better than the NC group. The two mice groups could be effectively distinguished using cluster analysis depending on tumor weight and AKR1B1 expression level. Therefore, AKR1B1 expression levels are critical in GC tumorigenesis and growth.

The AKT-mTOR signaling pathway modulates the migration, proliferation, metabolism, and drug resistance of cancer cells [14, 36]. The AKT-mTOR alteration may be affected by MAPK, AMPK, Hedgehog, and other signaling pathways [14, 37, 38]. The TCGA data bank was analysed by the GEPIA platform based on the numerous analogous influences of AKR1B1 and AKT-mTOR on cancer cells. The results revealed that the AKR1B1 gene level had positive correlation with the expression of AKT and mTOR in GC. Meanwhile, the expression of AKR1B1 was reduced, and changes in the phosphorylation level of the AKT-mTOR pathway were detected to demonstrate the regulatory role of AKR1B1 on this pathway at the protein level. The knockdown of AKR1B1 expression in GC cells could efficaciously inhibit AKT and S6K1 phosphorylation, confirming the regulation of the Akt-mTOR pathway by AKR1B1. Khayami et al. considered that the inhibition of AKR1B1 could decrease the AKT phosphorylation in small bowel and large bowel in Apc^Min/+^ mice with a high fat diet. Meanwhile, AKR1B1 inhibition could block the mTOR pathway activation by activating 5′ adenosine monophosphate-activated protein kinase (AMPK) as it inhibits the phosphorylation of mTOR, Raptor, eIF4E, S6K, and 4E-BP1, thus inhibiting tumor growth. These regulatory pathways can synthesize the regulatory effects of AKR1B1 on mTOR-related signaling networks [39, 40].

Abnormal elevated expression of AKR1B1 in GC could lead to poor prognosis among GC sufferers. AKR1B1 could be an independent prognostic factor for postoperative GC patients. The prognostic model established according to AKR1B1 expression depicted its considerable impact on patient prognosis. Additionally, *in vitro* and *in vivo* experiments showed that AKR1B1 could accelerate GC cell migration and proliferation ability while affecting the nutritional level by controlling the AKT-mTOR pathway. These findings may provide the theoretical foundation for confirming the prospective connection between AKR1B1 and AKT-mTOR pathway and its regulatory mechanism, providing novel strategies for targeted GC therapy.
